# Enhancing Antibiotic Efficacy and Combating Biofilm Formation: Evaluating the Synergistic Potential of *Origanum vulgare* Essential Oil against Multidrug-Resistant Gram-Negative Bacteria

**DOI:** 10.3390/microorganisms12081651

**Published:** 2024-08-12

**Authors:** Bilal Saoudi, Karim Bariz, Sarah Saci, Yousra Belounis, Hakima Ait Issad, Mohamed Abbaci, Mohamed Abou Mustapha, El-Hafid Nabti, Rawaf Alenazy, Mohammed Sanad Alhussaini, Abdulrahman A. I. Alyahya, Mohammed Alqasmi, Maryam S. Alhumaidi, Fawaz M. Almufarriji, Karim Houali

**Affiliations:** 1 Laboratory of Analytical Biochemistry and Biotechnology, Faculty of Biological and Agronomic Sciences, Mouloud Mammeri University of Tizi-Ouzou, Tizi Ouzou 15000, Algeria; saoub52@gmail.com (B.S.); brizk@yahoo.fr (K.B.); sarittas694@gmail.com (S.S.); belounisyousraw@gmail.com (Y.B.); 2 Laboratoire Ressources Naturelles, Mouloud Mammeri University of Tizi-Ouzou, Tizi Ouzou 15000, Algeria; hakima.aitissad@fs.ummto.dz; 3 Centre de Recherche Scientifique et Technique en Analyses Physico—Chimiques CRAPC, Bou Ismail 42004, Algeria; abbaci.mohamed@crapc.dz (M.A.); aboumustapha.mohamed@crapc.dz (M.A.M.); 4 Laboratoire de Maitrise des Energies Renouvelables, Faculté des Sciences de la Nature et de le Vie, Université de Bejaïa, Bejaïa 06000, Algeria; nabtielhafid1997@univ-bejaia.dz; 5 Department of Medical Laboratory, College of Applied Medical Sciences-Shaqra, Shaqra University, Shaqra 11961, Saudi Arabia; malhussaini@su.edu.sa (M.S.A.); alya7ya@su.edu.sa (A.A.I.A.); malqasmi@su.edu.sa (M.A.); 6 Department of Biology, College of Science, University of Hafr Al Batin, P.O. Box 1803, Hafr Al Batin 31991, Saudi Arabia; maryamalhumaidi@uhb.edu.sa; 7 Medical Laboratories Department, College of Applied Medical Sciences in Al-Quwayiyah, Shaqra University, Shaqra 11961, Saudi Arabia; falmufarriji@su.edu.sa

**Keywords:** antibacterial resistance, essential oil, *Origanum vulgare*, synergistic effect, antibiofilm activity, gram negative bacteria

## Abstract

Multidrug-resistant (MDR) Gram-negative bacteria remain a global public health issue due to the barrier imposed by their outer membrane and their propensity to form biofilms. It is becoming imperative to develop new antibacterial strategies. In this context, this study aims to evaluate the antibacterial efficacy of *Origanum vulgare* essential oil (OEO), alone and in combination with antibiotics, as well as its antibiofilm action against multidrug-resistant Gram-negative strains. OEO components were identified by gas chromatography-mass spectrometry (GC-MS), and antibacterial activity was assessed using the agar diffusion test and the microdilution method. Interactions between OEO and antibiotics were examined using the checkerboard method, while antibiofilm activity was analyzed using the crystal violet assay. Chemical analysis revealed that carvacrol was the major compound in OEO (61.51%). This essential oil demonstrated activity against all the tested strains, with inhibition zone diameters (IZDs) reaching 32.3 ± 1.5 mm. The combination of OEO with different antibiotics produced synergistic and additive effects, leading to a reduction of up to 98.44% in minimum inhibitory concentrations (MICs). In addition, this essential oil demonstrated an ability to inhibit and even eradicate biofilm formation. These results suggest that OEO could be exploited in the development of new molecules, combining its metabolites with antibiotics.

## 1. Introduction

Antibiotic resistance ranks among the ten gravest threats to humanity, and its steadily worsening antibiotic resistance is one of the 10 major threats to humanity and has been growing steadily for decades. In 2019, this phenomenon caused the death of 1.27 million people and indirectly contributed to the death of 4.95 million others [1]. Moreover, it is estimated that by 2050, these numbers will rise to ten million deaths a year, resulting in additional healthcare costs of one trillion dollars [2,3]. Although the dramatic rise in bacterial resistance is due to several factors, the inappropriate use of antibiotics remains one of its main causes [4].

The spread of this resistance is at the origin of the continuous emergence of multidrug-resistant bacteria. This phenomenon is particularly concerning for Gram-negative bacilli (GNB), which include *Enterobacteriaceae*, *Pseudomonas aeruginosa*, and *Acinetobacter baumannii*. These bacteria have several resistance mechanisms, including an outer membrane that hinders antibiotics, efflux pumps that remove drugs, and the production of enzymes that break down antibiotics, such as the highly prevalent extended-spectrum β-lactamases (ESBLs), particularly among Gram-negative bacilli (GNB). These mainly include *Enterobacteriaceae*, *Pseudomonas aeruginosa*, and *Acinetobacter baummanni*. The presence of an outer membrane and efflux pumps hinders the action of many antibiotics [5,6]. This is in addition to the fact that these bacteria develop increasingly sophisticated mechanisms, notably the production of enzymes such as extended-spectrum β-lactamases (ESBLs). The latter is the most frequent in clinical settings, conferring resistance to all classes of β-lactam except carbapenems [7].

Furthermore, this resistance has also affected the carbapenem class, following their overuse in the treatment of infections caused by ESBL-producing strains. The resistance mechanisms are often acquired through the horizontal transfer of mobile genetic structures, which also carry genes for resistance to other antibiotic classes such as fluoroquinolones and aminoglycosides. This situation leaves clinicians facing a real therapeutic impasse [8].

Resistance to treatments is further complicated by the ability of these bacteria to form biofilms on both living tissues (biotic) and inanimate surfaces (abiotic). These structures act as a real barrier, preventing the antibiotic from reaching their sites of action. Thus, these bacterial communities are 1000 times more resistant than planktonic populations [9]. Biofilms are generally associated with dental disease, endocarditis, deep-seated infections, and infections associated withindwelling devices or catheters. Management of such infections often requires prolonged hospitalization, surgery, the removal of infected implants, and postoperative antibiotic therapy, which further increases the cost of care [10]. Because of all these complications, the World Health Organization (WHO) has ranked these bacteria at the top of its list of priority germs for which new therapeutic approaches are urgently required [11].

Essential oils (EOs) are a promising source of new molecules with antibacterial and antibiofilm activity [12,13,14]. Remarkably, some reports have also revealed that even greater activity can be achieved by combining these EOs with certain antibiotics [15,16]. This combination strategy can resuscitate the efficacy of conventional antibiotics against resistant bacteria, which makes it extremely interesting, as it could bring back to the market antibiotics that have been abandoned due to their loss of efficacy, since the development of new molecules requires several years and substantial budgets [17,18].

*Origanum vulgare*, a medicinal plant from the *Lamiaceae* family, is particularly well-known in the Kabylie region of Algeria. It is mainly used in herbal teas to treat digestive and respiratory disorders [19]. The EO of this plant is one of the most studied oils worldwide thanks to its remarkable antimicrobial and antioxidant properties. These activities are attributed to the richness of this EO, particularly with carvacrol and thymol, as well as γ-terpinene and p-Cymene [20]. Several reports have thus revealed the potential of oregano oil both alone and in combination with antibiotics. However, the majority of studies have been limited to a single bacterial genus and/or antibiotic class [21,22]. To the best of our knowledge, no study has simultaneously reported the combined effects of this oil with different classes of antibiotics, and against various clinical bacterial genera. In this context, this study aims to determine the chemical composition of *Origanum vulgare* EO, and to evaluate its antibacterial and antibiofilm activity and its interactions with different conventional antibiotics against multidrug-resistant clinical Gram-negative strains.

## 2. Materials and Methods

### 2.1. Plant Collection

The *Origanum* plant was gathered from the locality of Iferhounene (36°32′11.4′′ N, 4°21′36.3′′ E), situated in the Kabylia region (Tizi-Ouzou state) of northern Algeria. Dr. Hocine Abbaci from the University of Bejaia conducted the botanical identification according to the Dobignard and Chatelain systematic guide from 2020. A sample was deposited at the herbarium of the El-Harrach National School of Agronomy-Algeria under the number 2023/NSA/40.

### 2.2. Essential Oil Extraction

To extract *Origanum vulgare* essential oil (OEO), the aerial parts of the plant were air-dried at room temperature for one week. Subsequently, the dried material underwent steam distillation using a Clevenger-type apparatus (Joanlab, Huzhou City, Zhejiang Province, China) for 3 h. The essential oil was collected through decantation, dehydrated using anhydrous sodium sulfate, and then stored in sealed dark glass vials at +4 °C until use [23].

### 2.3. Gas Chromatography-Mass Spectrometry (GC-MS) Analysis

The EO’s chemical composition was identified at the Center of Physico-Chemical Analyses in Tipaza, Algeria. GC-MS analyses were performed using a Hewlett Packard Agilent 6890 plus device equipped with an HP-5MS column (30 m, 0.25 mm, 0.25 µm) and a Hewlett Packard Agilent 5973 mass detector operating in ICT Scan mode (30 to 550) (Agilent Technologies, Santa Clara, CA, USA). Helium was used as a carrier gas at a 0.5 mL/min flow rate. The oven temperature program was as follows: 60 °C for 8 min, 2 °C/min up to 250 °C, and isothermal for 10 min. The detector temperatures were 280 °C. The identification of components was based on the comparison of their mass spectra and retention indices with those of NIST Libraries or with the literature [24].

### 2.4. Tested Bacterial Isolates

In this study, six bacterial strains were tested: two reference strains; *Pseudomonas aeruginosa* ATCC 27853, and *Klebsiella pneumoniae* ATCC 700603 ESBL-positive (SHV-18), and four clinical Gram-negative strains isolated from different units of the Tizi-Ouzou University Hospital; *E. coli* 45, *K. pneumonia* 5096, *P. aeruginosa* 150, and *Acinetobacter baumannii* 14889. These strains were identified by API 20^E^ gallery and then stored in glycerol at −18 °C until use.

### 2.5. Antibiotic Susceptibility Test

#### 2.5.1. Disk Diffusion Method

The susceptibility of the four clinical strains was tested using the agar diffusion method, against the following antibiotics: Amoxicillin, Amoxicillin-clavulanic acid, Ticarcillin, Ticarcilln-clavulanate, Cefazolin, Cefoxitin, Ceftriaxone, Ceftazidime, Cefotaxime, Imipenem, Gentamicin, Amikacin, Ciprofloxacin, Ofloxacin, Tetracycline, Fosfomycin, Trimethoprim-sulfamethoxazole, and Chloramphenicol. After incubation at 37 °C for 24 h, the diameters of the inhibition zones around the discs were measured. The antibiotic disc content and interpretation of the results were defined according to the Clinical Laboratory Standards Institute (CLSI-M100) guidelines [25].

#### 2.5.2. Determination of MICs of Antibiotics

The MIC values were determined for the most commonly used antibiotics, cefazolin, cefotaxime, ciprofloxacin and gentamicin, using the broth microdilution method in accordance with CLSI, M07-A9 [26]. Antibiotic stock solutions were prepared at 1024 µg/mLand ½ dilutions were carried out down to the concentration of 1 µg/mL. For some strains whose MICs were not detected within these ranges, concentrations ranging from 1024 to 8192 µg/mL were used.

### 2.6. Antibacterial Activity of Essential Oil

#### 2.6.1. Disk Diffusion Method

The bacterial suspension (10^8^ CFU/mL) was flood-inoculated on the surface of the Muller-Hinton (MH) agar (Oxoid) plate. Discs of Whatman No.1 paper (6 mm in diameter) were deposited on MH agar, and then 10 µL of EO were placed on each disc [16]. The plates were stored for 3 h at 4 °C, for the prediffusion of OEO in the agar [23], and then incubated at 37 °C for 24 h. The diameters of the inhibition zones around the discs were measured. All tests were performed in triplicate and the result was expressed as the mean of the three test results ± standard deviation.

#### 2.6.2. Determination of MICs and MBCs of Essential Oils

The minimum inhibitory concentration values of the essential oil were determined using the broth microdilution assay according to the CLSI, M07-A9 protocol [26]. Briefly, 50 µL of Mueller-Hinton Broth (MHB) (Oxoid) supplemented with 1.0% Tween 80 was dispensed from the second to the tenth well of a 96-well microplate. Subsequently, 100 µL of the essential oil (EO) was added to the first test well, and two-fold dilutions were made from the second to the tenth well, each containing 50 µL. Bacterial suspensions were prepared from 18-h cultures using saline, diluted in MHB, and 50 µL was added to all wells at a density of 10^6^ CFU/mL. The eleventh and twelfth wells served as controls, containing MHB supplemented with 1.0% Tween 80 and MHB, respectively. All the tests were conducted in triplicate. Following 18 h of incubation at 37 °C, readings were taken by adding 30 µL of resazurin (0.015%) followed by 3 h of incubation [23]. Resazurin reveals bacterial growth through the appearance of pink coloration.

To assess the MBCs, 10 µL of broth from the wells corresponding to MIC, 2 × MIC, and 4 × MIC values was transferred on the Muller-Hinton agar plate and incubated for 24 h at 37 °C. After incubation, the MBC was identified as the concentration on which no colony growth was observed [27].

### 2.7. Checkerboard Assay

The interactions between OEO and antibiotics were assessed using the checkerboard method, which consists of testing several concentrations of the antibiotic with various concentrations of the EO. Briefly, dilutions of OEO were performed vertically, into the 96-well microplates at decreasing concentrations, going from MIC × 2 to MIC/32. For antibiotics, the dilutions were carried out horizontally, at decreasing concentrations, going from MIC × 2 to MIC/256. Each well contained 25 µL of the EO, 25 µL of one of the antibiotics, and 50 µL of the bacterial suspension at 10^6^ CFU/mL. The plates were incubated at 37 °C for 24 h, and the bacterial growth was visualized by adding 30 µL of resazurin [23]. The type of interaction was determined by calculating the fractional inhibitory concentration index (FICI), using the following formula [28]:

FIC index = FICA + FICB

$$FICA=\frac{MIC\;of\;\left(A\right)\;in\;combination}{MIC\;of\;\left(A\right)\;alone}$$

$$FICB=\frac{MIC\;of\;\left(B\right)\;in\;combination}{MIC\;of\;\left(B\right)\;alone}$$

where (A) is *Origanum* EO and (B) is the antibiotic.

The interaction was considered synergistic when FICI ≤ 0.5, partially synergistic when 0.5 < FICI < 1, additive if FICI = 1, indifferent when >1 < FICI ≤ 4, and antagonistic when FICI > 4.

### 2.8. Biofilm Formation Test

The microtiter plate biofilm formation assay assessed the biofilm formation ability of the tested bacterial strains. A single colony was taken from the MHA overnight bacterial culture, inoculated into 0.9% saline solution, and adjusted to 10^6^ (CFU/mL) by diluting with TSB supplemented with 2% glucose (TSBG) [29]. We mixed 100 µL of (TSBG) with 100 µL of inoculum into a 96-well microplate. Negative control wells were filled with 200 µL of media only. After incubation of 48 h at 37 °C under static conditions, the microplate content was discarded by aspiration and each well was washed twice with 250 µL saline water 0.9% using a micropipette, to remove all the planktonic cells while preserving the integrity of the biofilm. After washing, the attached bacteria were left to dry for 60 min at 60 °C to promote biofilm fixation [30]. The remaining bacteria attached to the bottom of the wells were stained with 150 µL of crystal violet 1% and incubated at room temperature for 15 min. Subsequently, crystal violet was eliminated, and the excess stain was rinsed three times with sterile water. Finally, 150 µL of methanol (99%) was added to each well for 15 min at room temperature to promote the crystal violet solubilization that had already penetrated the cells. All the tests were performed in triplicate. The optical density (OD) was measured at 630 nm using a microplate reader. Non-inoculated TSB was used as a negative control and the cut-off value was calculated using the following formula [31]: ODc = Average OD of negative control + 3 SD (Standard Deviation) of negative control. Based on the cut-off OD calculated, strains were classified into the following categories: Non-biofilm producers (OD < ODc), weak biofilm producers (ODc < OD < 2 × ODc), moderate biofilm producers (2 × ODc < OD < 4 × ODc) and strong biofilm producers (OD > 4 × ODc).

### 2.9. Effect of OEO on Biofilm Adhesion and Preformed Biofilm

The action of OEO on biofilms was evaluated before and after its formation. Firstly, we studied the ability of OEO to prevent biofilm adhesion, and secondly, we investigated the ability of this oil to eradicate a preformed biofilm.

To inhibit biofilm adhesion by OEO, 100 µL of EO dissolved in TSBG was dispensed into each well. Bacterial suspensions (10^6^ CFU/mL) were prepared as described above and aliquots of 100 µL were added to all the wells. The final concentration of the EO was equivalent to MIC and the final volume was 200 µL. Untreated cells served as negative controls. After incubation at 37 °C for 24 h, the formed biofilm was quantified by using 1% crystal violet as described previously. All the assays were performed in triplicate [32].

To investigate the OEO’s ability to eradicate an established biofilm, a 48-h-old biofilm was developed in a 96-well microplate incubated at 37 °C. Each well was washed twice with sterile saline water 0.9%, and 200 µL of EO dissolved in TSBG was added to every well at a concentration equivalent to MIC. The performed biofilms, added with 100 µL of TSBG and 100 µL of sterile distilled water, served as controls. After incubation overnight at 37 °C, the density of the treated and untreated biofilms was quantified with the crystal violet procedure. All the assays were carried out in triplicates.

The percentage of biofilm inhibition or eradication was calculated by the comparison between the absorbance of untreated and treated biofilm according to the following formula [33]:

$$\%\;of\;inhibition\;or\;eradication=\frac{\left(OD\;growth\;control)-(OD\;sample\right)}{OD(growth\;control)}\times 100$$

### 2.10. Statistical Analysis

All the experiments were performed in triplicate, and the results were presented as the mean ± standard deviations (SD). The results of the antibacterial activity were subjected to the analysis of variance (ANOVA) with Tukey’s post-hoc test, using SPSS 25. Statistical significance was set at *p* < 0.05.

## 3. Results

### 3.1. Extraction and Chemical Composition of Essential Oil

The aerial parts of *O. vulgare* yielded an EO of 1.6% (*w*/*w*). The chemical compounds of this EO are listed in Table 1. The results showed a particularly high monoterpene content. The major compounds were carvacrol (61.51%), γ-terpinene (13.95%), and β-Cymene (8.56%), followed by constituents with lower levels such as; 4-carene (2.43%), β-pinene (1.68%), β-caryophyllene (1.66%), thujene (1.64%), and linalool (1.08%). Other components such as Eugenol, α-Pinene, 4-Terpineol, and Sabinene hydrate were present at levels below 1%.

### 3.2. Antibiotic Susceptibility Testing of Clinical Strains

The antibiogram results showed that all the bacterial strains collected were MDR, with resistance to at least three different classes of antibiotics (Table 2). The two *Enterobacteriaceae* strains showed a similar profile, with resistance to penicillins, 1st- and 3rd-generation cephalosporins, fluoroquinolones (CIP), and sulfonamides (SXT). *P. aeruginosa* 150 was only sensitive to CAZ, while *A. baumannii* 14889 was resistant to all the tested antibiotics.

### 3.3. Activity of Oreganum Essential Oil

*O. vulgare* essential oil showed activity against all *Enterobacteriaceae* strains with IZDs ranging from 16 ± 1.0 to 26.6 ± 1.1 mm and MIC values varying from 1.2 ± 0.5 to 2.35 ± 1.0 mg/mL. Moreover, sensitivity to OEO was not significantly different (*p* > 0.05) between these strains. *A. baumannii* 14889 was significantly (*p* < 0.05) more sensitive to the EO than the other strains, with an IZD of 32.3 ± 1.5 mm and MIC value of 0.88 ± 0.0. On the other hand, *P. aeruginosa* 150 and *P. aeruginosa* ATCC 27853 were considered as the most resistant to OEO with IZDs of 7.6 ± 0.5 and 12.3 ± 1.1 mm and MICs values of 7.03 ± 0.0 and 14.0 ± 0.0, respectively (Table 3).

### 3.4. Combination of O. vulgare EO with Antibiotics

**Figure 1:**
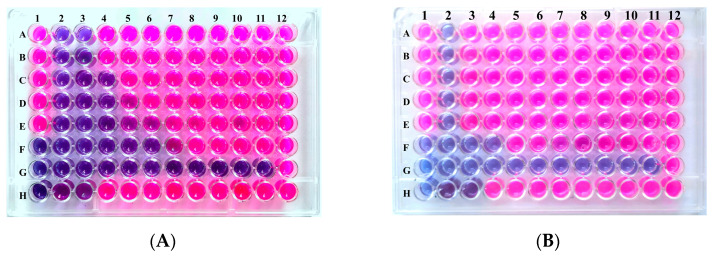
Checkerboard assays using OEO and antibiotics. Blue wells indicate growth inhibition. Pink wells indicate growth. H1 well is used for sterility control, and the 12th-column is for growth control. H2–H11 contains antibiotic alone, while G1–A1 contains the OEO alone. All the other wells contain combinations of antibiotic and OEO. (**A**) Synergistic combination between OEO and CIP against *A. baumannii* 14889 strain. (**B**) Additive effect observed between OEO and CTX against *E. coli* 45 strain.

It is important to note that these combinations can result in either a combined MIC (MIC_C_) equal to the individual MIC, or a MIC_C_ in which only one of the MICs (antibiotic or OEO) has decreased, or a MIC_C_ resulting from a decrease in both individual MICs at the same time. Our study revealed that combining OEO with GEN exerted a totally or partially synergistic effect against *E. coli* 45, *K. pneumoniae* 5096, and *P. aeruginosa* 150, respectively, with FICIs (fractional inhibitory concentration indexes) ranging from 0.38 to 0.75. Two further synergistic effects were observed when OEO and CTX were combined against *P. aeroginosa* ATCC 27853 and *A. baumannii* 14889, with FICIs of 0.27 and 0.28, respectively. The best synergistic combination was that of OEO and CIP against *A. baumannii* 14889, with an FICI of 0.12 (Figure 1A). In all these synergistic combinations, a reduction in MICs was reported, with rates reaching 98.44% for the antibiotic and 93.75% for OEO. Furthermore, additive effects (FICI = 1) were produced when OEO was combined with cephalosporins (CZ, CTX) against the clinical strains of *Enterobacteriaceae* (Figure 1B), or with CIP against *P. aeruginosa* 150, where the MICs of both agents decreased, with rates ranging from 50 to 75%. Finally, indifference was particularly demonstrated by all combinations against *K. pneumoniae* ATCC 700603, by the combination of OEO with CIP against other *Enterobacteriaceae* strains, and by that of OEO/GEN against *A. baumannii* 14889, with FICIs ranging from 1.25 to 2 and a reduction varying from 50 to 75% in OEO MICs (Table 4).

### 3.5. Biofilm Formation Test for Bacterial Strains

Bacterial strains were tested for their ability to form a biofilm on a polystyrene surface (Table 5). The results showed that all strains were biofilm producers, except *E. coli* 45. *A. baumannii* 14889 was a strong biofilm producer, with an OD of 1.385 ± 0.162, followed by *K. pneumoniae* ATCC 700603, *P. aeruginosa* ATCC 27853, and *P. aeruginosa* 150, which were moderately biofilm-forming, with ODs ranging from 0.944 ± 0.125 to 1.080 ± 0.106, whereas *K. pneumoniae* 5096 was weakly formative with an OD of 0.372 ± 0.101.

### 3.6. Antibiofilm Activity of Origanum Essential Oil

**Figure 2:**
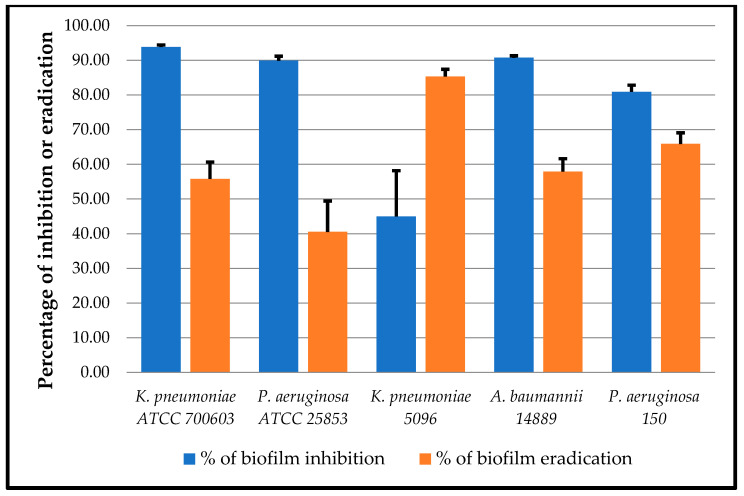
Percentages of inhibition of biofilm formation (blue) and eradication of preformed biofilm (orange) by OEO against tested bacterial strains.

The antibiofilm activity of OEO was evaluated against biofilm-producing strains, by measuring the percentages of inhibition of biofilm formation and eradication of preformed biofilms. The results in Figure 2 showed that OEO, at MICs-equivalent concentrations, was able to inhibit biofilm formation in all strains, with inhibition percentages ranging from 44.98 to 93.83%. Furthermore, OEO had an eradication activity against all preformed biofilms, with percentages ranging from 40.52 to 85.32%.

## 4. Discussion

The GC-MS analysis revealed a rich composition of the EO, dominated by oxygenated monoterpenes (e.g., carvacrol), followed by hydrogenated monoterpenes such as γ-terpinene and β-Cymene, which is a common feature of oregano EOs [34]. However, it stands out for the absence of thymol, although it contrasts with findings from other studies, such as those by Amrouni et al. [35] and Giamperi et al. [36], who reported significant levels of thymol (23.64% and 17%, respectively) alongside carvacrol (33.85% and 33.4, respectively) in oregano EO from different regions (Guelma and Italy). On the other hand, some recent studies, such as that of Ebani et al. [37], have reported chemical profiles without thymol and with 65.9% of carvacrol, which is qualitatively similar to our results.

The great variability in the chemical composition of EOs could be explained by several factors, such as the part of the plant used (leaves, flowers, etc.), the geographical area and the altitude of the plant. Indeed, Moisa et al. [38] revealed that the main compound in EO extracted from oregano leaves was linalool (23.91%), while in EO extracted from flowers it was γ-terpinene (29.26%). The influence of the geographical area on EO composition was demonstrated by Goyal et al. [39], who observed that in India, EO from Pithoragarh was richer in thymol (52.83%) than that from Auli (38.81%), given that these two regions are located in the north-east of the country, 324 km apart. Regarding altitude, Öner and Yeşil [40] noted that the carvacrol content in EOs increased with plant altitude, reaching a maximum percentage of 21.50% at 1387 m altitude. This may explain our results, since our plant was located in a mountainous region at an altitude of 1300 m. In view of this composition, our EO is of the “cymyl” type, which is the most economically important chemotype, as the carvacrol it contains is widely used in the food and cosmetics industry [41].

In the context of antibiotic resistance, bacteria are classified as MDR (multidrug-resistant) when they resist at least three different antibiotic classes [42]. By this definition, all the clinical strains tested in our study were MDR. The resistance patterns observed in these strains were similar to those reported in some studies in Algeria and other countries [43,44,45,46,47]. These resistances likely stem from two factors: the overuse of certain antibiotics (e.g., cephalosporins and fluoroquinolones) and the reliance on last-resort antibiotics (e.g., imipenem, amikacin) for severe infections, given the limited treatment options available in Algeria. This situation creates constant pressure for research laboratories to develop new therapeutic alternatives.

*O. vulgare* essential oil seems to be of great interest against such bacteria, given the results reported by certain studies. Nabti et al. [48] revealed that five OEOs from different Algerian regions were active against MDR uropathogenic *E.coli* strains, with IZDs ranging from 24.6 ± 1.27 to 39.6 ± 0.77 mm. In addition, Mohsen et al. [49] reported the activity of an OEO from Iraq against the MDR *K. pneumoniae* strain with an IZD of 23 mm, and more recently, Silva et al. [22], in Brazil, demonstrated the remarkable efficacy of OEO against several MDR *K. pneumoniae* serotypes, with MICs values below 128 µg/mL. These results are thus qualitatively in agreement with those of our study. Concerning non-fermentative GNB, *A. baumannii* 14889 was highly sensitive to our EO, which is consistent with the study of Amaral et al. [21], which reported MICs ranging from 1.75 to 3.50 mg/mL of OEO against carbapenemase-producing *A*. *baumannii* isolates. In contrast, the two *P. aeruginosa* tested in this study were the least sensitive to OEO. These results were comparable to those found by Amrouni et al. [35], who noted that MDR strains of *P. aeruginosa* were more resistant than *E. coli* and *S. aureus* to OEO. Similarly, Abu Ghazal et al. [50] reported a marginal activity (IZD = 8 mm) of oregano oil against *P. aeruginosa* ATCC 27853.

The variable sensitivity of the bacterial strains tested in our study to EO could be attributed to the presence of differences in their outer membrane, notably in terms of size, number of LPS molecules, thickness, surface charge distribution, and dynamics, which in turn result in variability in permeability properties [51]. In addition, certain bacterial genera possess efficient specific efflux mechanisms, which confer intrinsic resistance to EO components, such as the MexAB-oprM system in *Pseudomonas* spp. [52]. Other studies have shown that sensitivity to EOs can vary, even between strains of the same species [53,54], making it more difficult to predict bacterial sensitivity to EOs and understand the factors on which it depends.

The mechanism of action of EOs is strictly dependent on the quality and quantity of their chemical compounds. In fact, their antibacterial activity does not result from a single mechanism, but rather from a cascade of reactions involving the entire bacterial cell [55]. However, it is generally accepted that the hydrophobicity of EO compounds enables them to interfere with the outer membrane lipids and interact with transmembrane proteins, thereby affecting cell permeability. In addition, these compounds can alter the process of energy generation, thus disrupting various cellular functions [56]. In general, this activity is attributed to the major constituents of EOs, as they have shown equivalent or even better effects than crude EOs when tested separately. Indeed, in the case of *O. vulgare* EO, Tapia-Rodriguez et al. [57] observed that the activity of carvacrol alone was better, with a MIC of 0.3 mg/mL, than that of crude oil, which had an MIC of 0.6 mg/mL against *A. baumannii* ATCC 19606. This remarkable efficacy of carvacrol has been explained by the presence of a hydroxyl group on its aromatic ring, which allows it to act as a proton exchanger across the cell membrane, leading to disruption of the proton motive force and hence ATP generation [58]. This suggests that the activity of our EO is mainly due to its high carvacrol content. However, it should be noted that its activity may be affected by interactions with other EO compounds. For example, P-Cymene may act synergistically with carvacrol, causing membrane swelling due to its high hydrophobicity, which facilitates the transport of phenolic compounds to the cytoplasm [55].

The combination of EOs with antibiotics is one of the most recent and widely considered strategies for solving the problem of bacterial resistance. Different effects can result from this combination, such as a synergistic effect when the combined activity is greater than the sum of the individual effects of the combined agents, an additive effect when a combination produces an effect equal to the sum of the individual effects, or an indifferent effect when there is no interaction between the two agents. Finally, an antagonistic effect can occur when the combined activity is reduced in comparison with the individual effects [18]. The aim of this approach is therefore to achieve combined effects between the EO and the antibiotic, reducing their concentrations while increasing antibacterial efficacy. Indeed, numerous studies have reported synergistic interactions between EOs and conventional antibiotics against Gram-negative bacteria. Iseppi et al. [59] observed that *Eucalyptus globulus* EO acted synergistically with CTX against clinical isolates of *E. coli*. In addition, Bariz et al. [23] reported the synergistic effect of *Thymus algeriensis* EO with amoxicillin-clavulanic acid, against *K. pneumoniae* ATCC 700603 and two ESBL-producing *K. pneumoniae* isolates. Abdelatti et al. [60] showed that cinnamon EO was synergistic with CIP against 83.33% of clinical strains of *P. aeruginosa.* Similar results were obtained in our study by combining OEO with GEN, CTX, and CIP. Interestingly, all these effects were accompanied by a considerable reduction in antibiotic MICs. This is very useful in therapy, to reduce their side effects, as well as the risk of the emergence of new bacterial resistances.

One of the most recognized mechanisms that could lead to this synergistic effect between EOs and intracellular-targeted antibiotics such as GEN and CIP is the destabilization of the outer membrane by EO components, which facilitates the penetration of these antibiotics towards the cytoplasm and better reaches their targets [61]. With β-lactam antibiotics (CTX), on the other hand, this synergy could be due to the ability of certain EO components to inhibit resistance mechanisms such as efflux pumps and β-lactamses, thus allowing the antibiotic to remain intact in the bacterium [12,62].

Research is indeed focusing more on synergistic effects in these combinations, but additive effects can also be interesting, as they produce satisfactory activity at lower antibiotic concentrations. In this study, the additive effects observed were all followed by a 50% decrease in antibiotic MICs. Similar effects were observed by Yang et al. [63], when combining oregano or peppermint EO with meropenem against *K. pneumoniae* BAA-1705.

In the end, predicting the effect of a combination will depend on the interactions of the different compounds of this EO with each other on the one hand, and with the antibiotic on the other. Moreover, the nature of the chemical groups associated with these molecules will be a determining factor in the type of action produced. This is the case, for example, for thymol and carvacrol isolated from oregano EO, which produced different effects when combined with penicillin. Indeed, this antibiotic combined with thymol showed excellent synergy against *E. coli*, while its combination with carvacrol showed indifference, even though chemically these two compounds differ only in the position of their hydroxyl group [64].

Another important feature of our EO, revealed in this study, is its ability to prevent biofilm formation and also to eradicate it after its establishment. The ability of bacteria to produce a biofilm depends on several factors linked to the bacteria themselves and to external environmental conditions (pH, temperature, surface…) [65]. In this study, all the tested strains formed biofilms at varying densities, except *E. coli* 45. Since the external conditions were identical for all these strains, this variability could be explained mainly by their different origins and the specificity of their virulence factors [66,67]. In addition, some studies have suggested a correlation between biofilm-forming capacity and resistance to specific antibiotics [68,69], but this aspect is subject to much divergence in the scientific literature.

The evaluation of the antibiofilm activity of *O. vulgare* EO revealed that this oil was highly effective, with inhibition percentages of up to 93.83%. These results were better than those reported by Lagha et al. [32], where the inhibition rates of uropathogenic *E. coli* biofilms did not exceed 88.21%. This activity was attributed to the ability of EO components to interfere with the various processes involved in biofilm formation, such as adhesion, bacterial mobility, and quorum sensing (QS). Indeed, biofilm formation requires several steps, the first of which is the reversible adhesion of bacteria to the surface. Carvacrol has been shown to interfere with this phase by intercalating into bacterial membranes, destabilizing membrane proteins and thus reducing cell attachment [70]. The other phenomenon crucial to biofilm structuring is mobility. This is ensured by flagella and pili, which enable bacteria to reach a specific site to colonize it. Mobility has also been affected by the effect of EOs, as observed in studies such as that of Merghni et al. [71], where the migration of *P. aeruginosa* was inhibited by *O. vulgare* EO. This inhibition is attributed, in fact, to the disruptive effect of EO on the QS system, which is a communication mechanism that controls mobility and all other processes involved in biofilm formation, through the expression of various virulence factors (EPS, andesins, etc…). Thus, Yuan et al. [72] observed that carvacrol significantly reduced the expression of genes encoding flagellar constituents and those controlling their rotation, leading to loss of mobility in *E. coli* O157:H7. In addition, the same compound was shown to inhibit EPS synthesis in *P. carotovorum* by acting directly on glucosyltransferase, an enzyme involved in the polymerization of sugars, or QS intermediates [73].

The eradication of preformed biofilms is more complex, especially with conventional treatments. Nevertheless, our EO was shown to reduce the density of 48-h-old biofilms, with rates reaching 85.32%. Similar effects were reported by Ben Abdallah et al. [74], where oregano EO eradicated 98.01% of *S. aureus* biofilm. One of the mechanisms that could be attributed to this effect is the ability of certain components such as carvacrol and thymol to diffuse through the polysaccharide matrix of the biofilm, enabling them to disrupt the biofilm and reach bacterial cells [75]. These results therefore demonstrate the value of using oregano oil in the fight against bacterial biofilms. However, further studies are required to confirm these effects against biofilms formed under in vivo conditions.

## 5. Conclusions

The continuing evolution of bacterial resistance in recent years has led to a search for new antibacterial molecules as alternatives to antibiotics. In this respect, *O. vulgare* EO from the Kabylie region of Algeria is active against MDR Gram-negative strains belonging to the most clinically dreaded genera. Furthermore, its combination with certain conventional antibiotics was observed to be of great interest, as it in no way affected their activity, but rather made it possible to reduce the antibiotic concentrations required against these bacteria. The evaluation of the antibiofilm activity of this EO revealed that inhibiting biofilm formation and eradicating could both inhibit biofilm formation and eradicate a large proportion of preformed biofilms. As a result, this EO could well be useful as an alternative to antibiotics, or at least as an adjuvant that could restore the use of conventional antibiotics against resistant bacteria. However, further studies are needed, on the one hand to understand the mode of action of this EO and the contribution of its constituents to its interactions with antibiotics, and on the other, to determine its toxicity threshold in vivo, with a view to its therapeutic use.

## Figures and Tables

**Table 1:** Chemical composition of *O. vulgare* essential oil.

No.	RI	RT	Compounds	Area (%)
1	844	5.231	Hexenal	0.04
2	923	8.758	Thujene	1.64
3	928	9.055	α-Pinene	0.75
4	940	9.895	Camphène	0.10
5	969	11.770	L-β-Pinene	0.16
6	983	12.713	Octen-3-ol	0.26
7	988	13.039	3-Octanone	0.09
8	992	13.330	β-Pinene	1.68
9	1000	13.879	α-Phellandrène	0.30
10	1005	14.245	(R)-α-Pinene	0.12
11	1013	14.845	4-Carene	2.43
12	1024	15.594	β-Cymene	8.56
13	1049	17.503	β-Ocymene	0.11
14	1060	18.314	γ-terpinene	13.95
15	1066	18.703	Sabinene hydrate	0.36
16	1084	20.057	Terpinolene	0.17
17	1103	21.446	Linalol	1.08
18	1136	23.812	o-Xylene	0.04
19	1145	24.481	Ethanone, 1-(1,4-dimethyl-3-cyclohexen-1-yl)	0.07
20	1160	25.596	Borneol	0.15
21	1172	26.476	4-Terpineol	0.52
22	1187	27.556	Terpineol	0.07
23	1207	28.951	p-menth-1-en-8-ol	0.35
24	1233	30.780	Thymol methyl	0.12
25	1242	31.397	Isothymolmethylether	1.27
26	1274	33.546	Carvone	0.10
27	1294	34.963	Ethanone	0.08
28	1326	37.038	Carvacrol	61.51
29	1360	39.210	Eugenol	0.84
30	1375	40.147	Cuminol	0.09
31	1409	42.308	β-Caryophyllène	1.66
32	1504	48.075	β-Bisabolene	0.21
33	1518	48.892	β-Sesquiphellandrene	0.13
34	1528	49.435	Eugenolacetate	0.14
35	1539	50.081	Humulene	0.07
36	1570	51.847	Caryophyllene oxide	0.33
			Monoterpenes hydrocarbons (MH)	29.85
			Oxygenated monoterpenes (OM)	65.43
			Sesquiterpenes hydrocarbons (SH)	2.07
			Oxygenated sesquiterpenes (OS)	0.33
			Others	1.49
			Total	99.17

RT: retention time, RI: retention indices relative to homologous n-alkanes C8–C24 obtained on an HP-5MS column.

**Table 2:** Results of antibiotics resistant profile of the collected bacterial isolates.

Antibiotic	*E. coli* 45	*K. pneumoniae* 5096	*A. baumannii* 14889	*P. aeruginosa* 150
AMX	R	R	N	N
AMC	R	I	N	N
TIC	N	N	R	R
TCC	N	N	R	R
CZ	R	R	N	N
FOX	S	S	N	N
CTX	R	R	R	N
CTR	R	R	R	R
CAZ	R	R	R	S
IMP	S	S	R	R
GEN	R	S	R	R
AK	S	S	R	R
CIP	R	R	R	R
OFX	R	N	R	R
TET	S	R	R	N
SXT	R	R	R	R
CHL	S	S	N	N
FOS	S	S	N	R

Sensible (S), intermediate (I), resistant (R), not done (N), amoxicillin (AMX), amoxicillin-clavulanic acid (AMC), ticarcillin (TIC), ticarcillin-clavulanic acid (TTC), cefazolin (CZ), cefoxitin (FOX), cefotaxime (CTX); ceftriaxone (CTR); ceftazidime (CAZ), imipenem (IMP), gentamicin (GEN), amikacin (AK), ciprofloxacin (CIP), ofloxacin (OFX), tetracycline (TET), trimethoprim-sulfamethoxazole (SXT), chloramphenicol (CHL), fosfomycine (FOS).

**Table 3:** Antibacterial activity expressed as IZD, MIC and MBC of OEO.

Bacterial Strains	IZD (mm)	MIC (mg mL^−1^)	MBC (mg mL^−1^)
*K. pneumoniae* ATCC 700603	16 ± 1.0	2.35 ± 1.0	4.6 ± 2.0
*P. aeruginosa* ATCC 27853	12,3 ± 1.1	14.0 ± 0.0	56.2 ± 0.0
*E. coli* 45	26.6 ± 1.1	1.76 ± 0.0	2.9 ± 1.0
*K. pneumoniae* 5096	17.6 ± 0.5	1.2 ± 0.5	4.6 ± 2.0
*A. baumannii* 14889	32.3 ± 1.5	0.88 ± 0.0	>3.52
*P. aeruginosa* 150	7.6 ± 0.5	7.03 ± 0.0	>28.1

**Table 4:** Combination testing of OEO (mg/mL) with antibiotics (µg/mL) against tested strains.

Strains	Combination	Individual MIC	Combined MIC	Individual FIC	FICI	Effect	MIC Reduction (%)
*K. pneumoniae* ATCC 700603	CZ/OEO CTX/OEO GEN/OEO	128/2.4 8/2.4 8/2.4	128/1.2 8/1.2 8/1.2	1/0.5	1.5	I	0/50 0/50 0/50
*P. aeroginosa* ATCC 27853	CTX/OEO	16/14.06	0.25/3.52	0.016/0.25	0.27	S	98.44/93.75
*E. coli* 45	CZ/OEO CTX/OEO GEN/OEO CIP/OEO	2048/1.76 2048/1.76 32/1.76 64/1.76	1024/0.88 1024/0.88 4/0.44 64/0.88	0.5/0.5 0.5/0.5 0.125/0.25 1/0.5	1.00 1.00 0.38 1.5	A A S I	50/50 50/50 87.5/75 0/50
*K. pneumoniae* 5096	CZ/OEO CTX/OEO GEN/OEO CIP/OEO	2048/1.2 128/1.2 32/1.2 16/1.2	1024/0.6 64/0.6 1/0.6 16/1.2	0.5/0.5 0.5/0.5 0.031/0.5 1/1	1.00 1.00 0.53 2.00	A A PS I	50/50 50/50 96.88/50 0/0
*A. baumannii* 14889	CTX/OEO GEN/OEO CIP/OEO	1024/0.88 4096/0.88 256/0.88	32/0.22 4096/0.22 16/0.055	0.031/0.25 1/0.25 0.062/0.062	0.28 1.25 0.12	S I S	96.88/75 0/75 93.75/93.75
*P. aeruginosa* 150	GEN/OEO CIP/OEO	128/7.03 32/7.03	64/1.76 16/3.52	0.5/0.25 0.5/0.5	0.75 1.00	PS A	50/75 50/75

FIC: fractional inhibitory concentration, FICI: fractional inhibitory concentration index, A: additive, I: indifference, S: synergistic, PS: partially synergistic.

**Table 5:** Biofilm formation test for the bacterial strains.

Strains	OD630 ± SD	Biofilm Formation
*K. pneumoniae* ATCC 700603	0.944 ± 0.125	Moderate biofilm producer
*P. aeruginosa* ATCC 27853	1.062 ± 0.054	Moderate biofilm producer
*E. coli* 45	0.240 ± 0.011	Non-biofilm producer
*K. pneumoniae* 5096	0.372 ± 0.101	Weak biofilm producer
*A. baumannii* 14889	1.385 ± 0.162	Strong biofilm producer
*P. aeruginosa* 150	1.080 ± 0.106	Moderate biofilm producer

OD: optical density, SD: standard deviation.

## Data Availability

The original contributions presented in the study are included in the article, further inquiries can be directed to the corresponding authors.
